# Congenital Partial Absence of Pericardium: A Mimic of Arrhythmogenic Right Ventricular Cardiomyopathy

**DOI:** 10.1155/2018/4297280

**Published:** 2018-04-10

**Authors:** J. S. Foo, C. H. Koh, A. Sahlén, H. C. Tang, C. P. Lim

**Affiliations:** ^1^Department of Cardiology, National Heart Centre, Singapore; ^2^Karolinska Institutet, Stockholm, Sweden

## Abstract

Congenital absence of pericardium is a rare condition with electrocardiogram, chest X-ray, and echocardiographic findings which may mimic those of other cardiac conditions. We present a case of a 19-year-old asymptomatic female with incidental cardiomegaly on chest X-ray and electrocardiographic and echocardiographic changes, which meet the revised task force criteria for definite arrhythmogenic right ventricular cardiomyopathy but subsequently confirmed to have congenital partial absence of pericardium on cardiac magnetic resonance imaging.

## 1. Introduction

**Figure 1:**
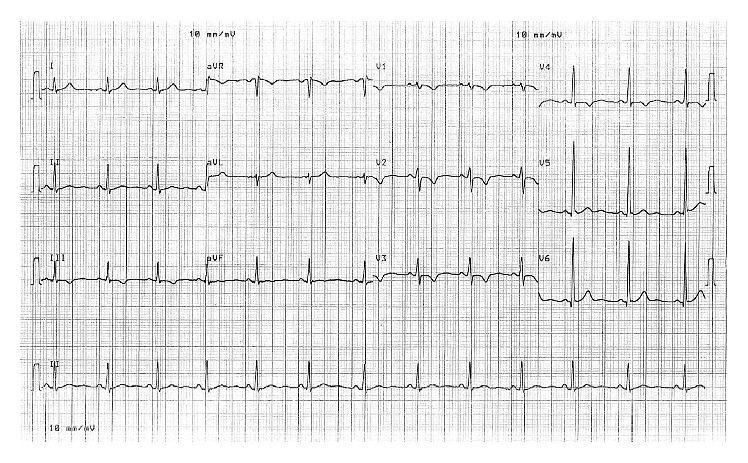
ECG showing T-wave inversions V1–V4.

**Figure 2:**
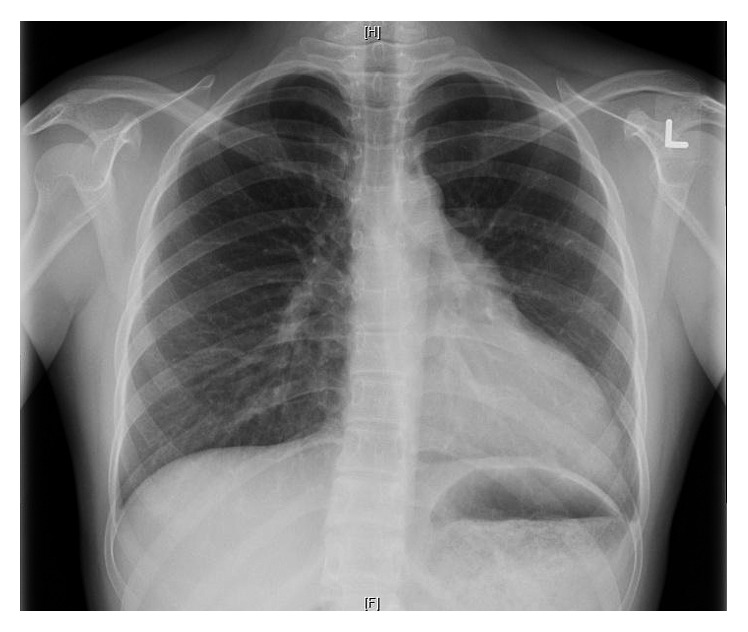
Chest X-ray demonstrating the Snoopy Dog Sign and lung tissue between the diaphragm and inferior border of the heart.

A 19-year-old Chinese female student was referred to our institution for abnormal T-wave inversions on the precordial leads of a screening electrocardiogram (ECG) (Figure 1), as well as a chest X-ray (CXR) showing cardiomegaly (Figure 2). These investigations were performed as part of routine preenrollment health screening for a local university course. She had no significant past medical history of note, nor any family history of sudden cardiac death or inheritable cardiac diseases. She had no cardiac symptoms and was physically active.

**Figure 3:**
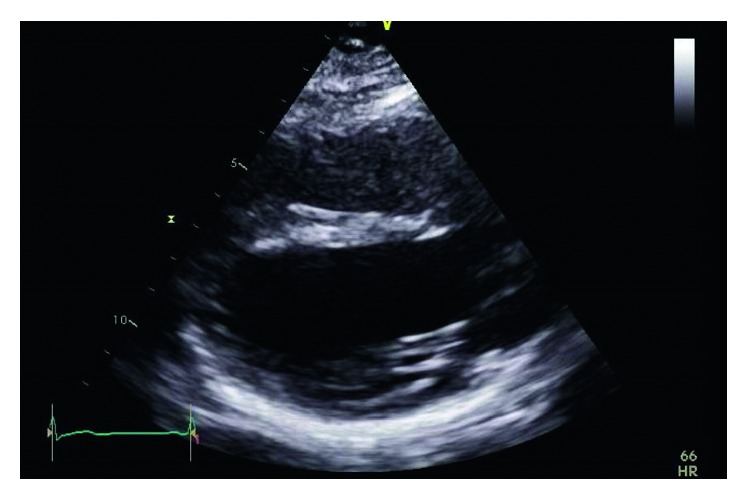
Dilated right ventricle on echo parasternal long view.

**Figure 4:**
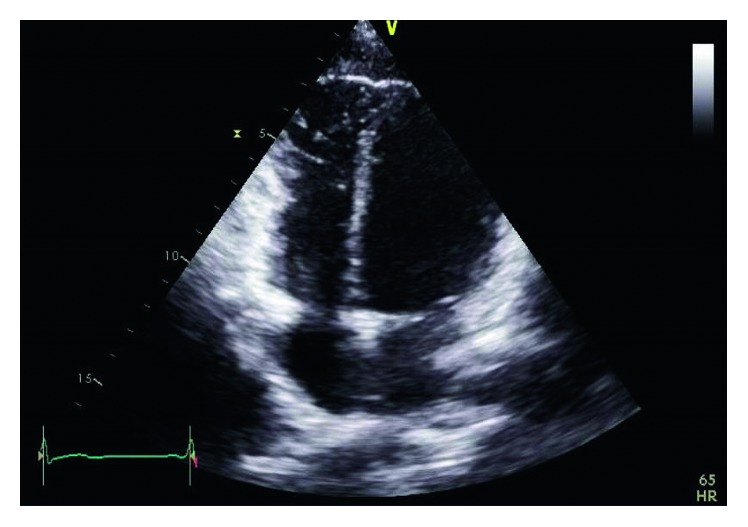
Echo showing bulging right ventricle suggestive of right ventricular aneurysm.

**Figure 5:**
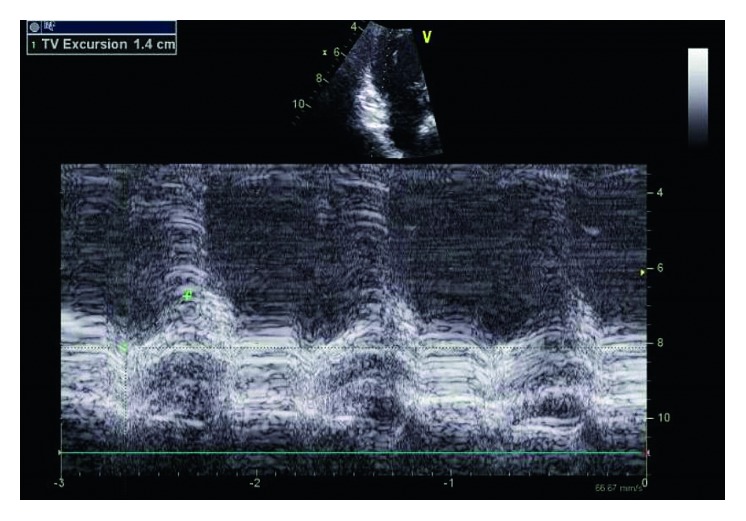
TAPSE of 1.4 cm.

**Figure 6:**
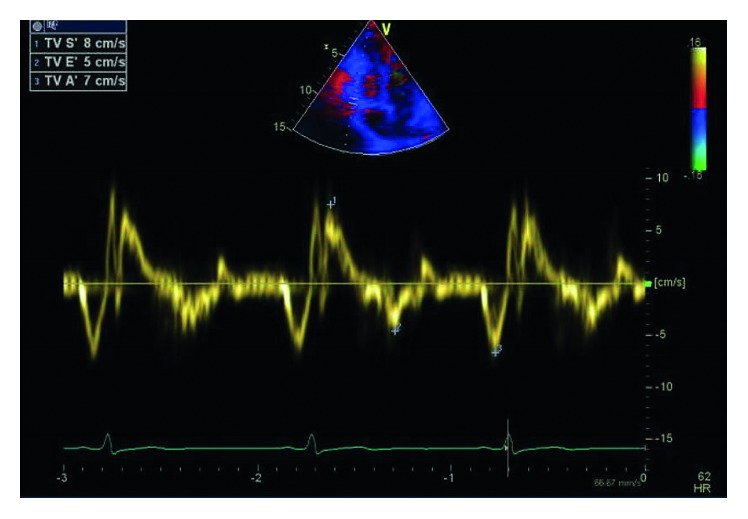
Tricuspid annulus tissue Doppler S wave of 0.08 m/s.

Physical examination was unremarkable. A repeat ECG showed symmetrical T-wave inversion in leads V1–V4. No epsilon waves were observed. An exercise stress echocardiogram was ordered, which returned negative for inducible myocardial ischemia at 95% maximum predicted heart rate after an exercise duration of 12 minutes (achieving 13.3 METs). However, a baseline echocardiography showed a visually and quantitatively impaired right ventricular function with tricuspid annular plane systolic excursion (TAPSE) of 1.4 cm and tricuspid annulus tissue Doppler S wave of 0.08 m/s. A focal segment of the distal right ventricular free wall appeared to be dyskinetic and outbulging, suggestive of right ventricular aneurysm. Left ventricular ejection fraction was normal at 69% with normal valves and atrial dimensions (Figures 3–6).

**Figure 7:**
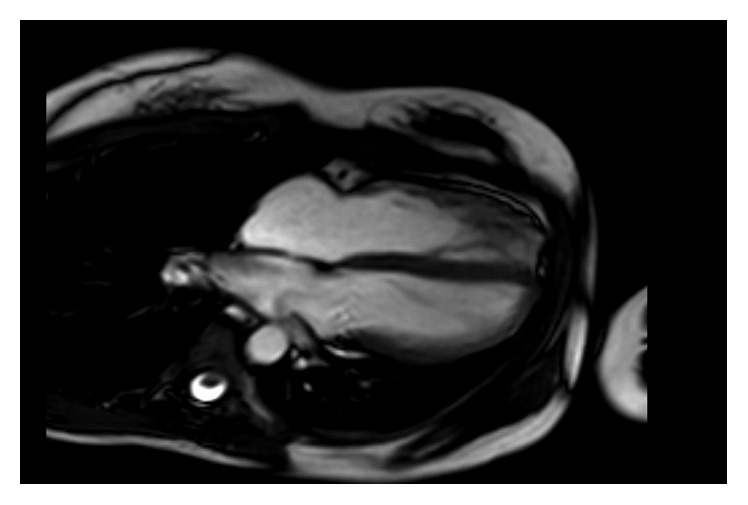
MRI cardiac showing inferoposterior position of the heart.

**Figure 8:**
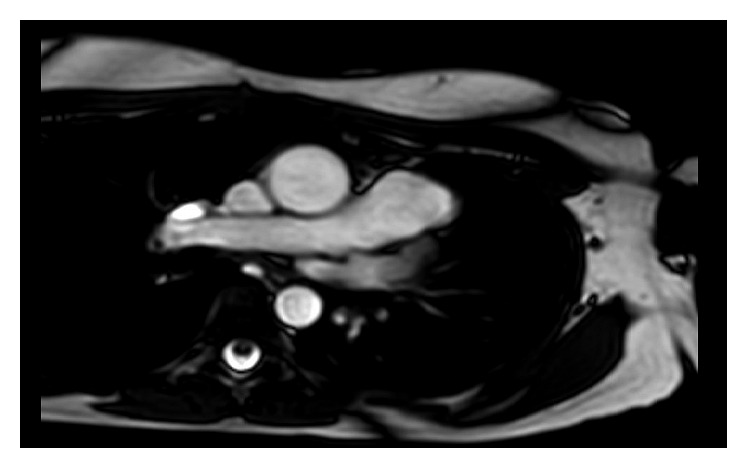
MRI cardiac showing interposition of lung tissue between aorta and pulmonary artery.

**Figure 9:**
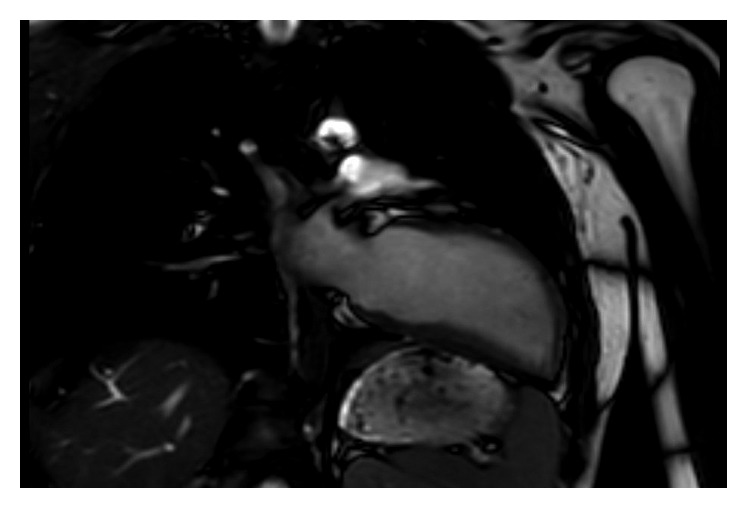
MRI cardiac showing typical teardrop shape of the heart with lung tissue between diaphragm and base of the heart.

In view of the findings suggestive of possible arrhythmogenic right ventricular cardiomyopathy (ARVC), a cardiac magnetic resonance imaging (MRI) was performed. Cardiac MRI without contrast showed right-sided pericardium but no pericardium present over the left heart, with characteristic displacement of the heart into the left hemithorax (Figures 7–9). There was normal right ventricular volumes and function (RVEDVi 86 ml/m^2^, RVESVi 35 ml/m^2^, and RVEF 60%) with no regional wall abnormality, specifically no dyskinetic or aneurysmal segment of the right ventricle. There were no structural abnormalities or entrapment of any epicardial coronary vessel at the edges of the defect. The patient was informed of the diagnosis of congenital partial absence of pericardium and discharged from follow-up.

## 2. Discussion

The normal pericardium is an avascular sac consisting of 2 layers: an outer fibrous layer and an inner serosal layer. Serous fluid normally occupies the space between the 2 layers and acts as a lubricant. With its ligamentous attachments, the pericardium stabilizes and maintains the position of the heart in the thorax.

Congenital absence of pericardium is a rare condition with an incidence of less than 1 in 10,000 [1]. Left-sided defects are the most common with a prevalence of 70% of all pericardial defects [2]. Complete bilateral absence of the pericardium makes up 9%, and right-sided defects compromise 17% of all defects [2, 3]. 30–50% of patients with congenital absence of pericardium have other congenital abnormalities, including atrial septal defect, patent ductus arteriosus, and tetralogy of Fallot [4]. Pericardial defects have also been seen in patients with aortic connective tissue disorders and Marfan syndrome [5, 6].

Congenital absence of pericardium is generally benign but can be confused with other pathologic conditions on basic imaging and screening tools. CXR commonly shows marked levoposition of the cardiac silhouette, loss of the right heart border, prominent pulmonary artery, and lung tissue between the diaphragm and inferior border of the heart [7]. On ECG, there is often poor R-wave progression due to axial rotation of the heart [7], and on echocardiography, there is right ventricular predominance due to the leftward displacement of the heart; as a result, the patient might be falsely labelled as having right ventricular dilatation [8, 9].

Patients with partial pericardial defects can be symptomatic with chest pain noted in one-third of patients [10]. They are also at risk of complications resulting from strangulation of any herniating cardiac structures (left ventricle, left atrium, and left atrial appendage) through the defect [11] and compression of coronary arteries [12], for which they will require surgical correction. Surgical pericardioplasty (Gore-Tex mesh) may be considered for highly symptomatic patients [13]. On the contrary, complete absence of pericardium does not commonly cause complications or symptoms. Patients might experience chest pain secondary to tension from pleuropericardial adhesions, lack of pericardial cushioning, and undue torsion or strain on the great vessels; as without a pericardium, they serve as the only anchor for the heart [2, 14].

In the case of our patient, electrocardiographic T-wave inversions in precordial leads, and the abovementioned echocardiographic findings, raised a suspicion of ARVC. A final diagnosis of partial absence of pericardium was reached by cardiac MRI.

In retrospect, after the diagnosis was reached on cardiac MRI, the chest X-ray of this patient was reviewed and found to be positive for the Snoopy Dog Sign with findings of levoposition of the cardiac silhouette and loss of the right heart border. There was a prominent pulmonary artery, lung tissue noted between the diaphragm and inferior border of the heart, and no right ventricular enlargement to suggest ARVC. The echocardiographic features of right ventricular outbulging and dyskinesia were due to leftward displacement of the heart.

Our patient has definite ARVC going by the revised task force criteria, meeting major criteria for echocardiographic changes and electrocardiogram repolarisation abnormalities. A diagnosis of ARVC implies a lifetime of follow-up, investigations, family screening, and potentially dangerous therapy such as implantable defibrillator. The cardiac MRI enabled the managing clinician to discharge the patient with no follow-up. A patient unfortunate enough to present with these findings in a unit with no cardiac MRI might have been labelled with a diagnosis of ARVC.

This case demonstrates the important role of cardiac MRI in cases with suspected ARVC, as changes on ECG and echocardiography are by no means pathognomonic for this disorder. In fact, other conditions manifesting with right ventricular dilatation may also mimic ARVC. We might have to take a closer look at ARVC registries for patients who have been wrongly included with congenital absence of pericardium.

In conclusion, while congenital absence of pericardium may be rare, this case emphasizes its importance in the differential diagnosis when evaluating electrocardiographic and echocardiographic changes suggestive of arrhythmogenic right ventricular cardiomyopathy.
